# Diagnosis of Granulomatosis With Polyangiitis in a 39‐Year‐Old Woman With a Recent History of Traveling to Malaria‐Endemic Region: A Case Report

**DOI:** 10.1002/ccr3.70327

**Published:** 2025-03-20

**Authors:** Hanie Forouzandeh, Ahmadreza Rajabi, Abbas Ali Torfeh Esfahani, Farzin Khorvash, Mansoor Karimifar

**Affiliations:** ^1^ School of Medicine Isfahan University of Medical Sciences Isfahan Iran; ^2^ Department of Infectious Diseases and Tropical Medicine, School of Medicine Isfahan University of Medical Sciences Isfahan Iran; ^3^ Department of Rheumatology Alzahra Hospital, Isfahan University of Medical Sciences Isfahan Iran

**Keywords:** autoimmune disease, granulomatosis with polyangiitis, infectious disease, Wegener's granulomatosis

## Abstract

Granulomatosis with Polyangiitis (GPA) is a rare vasculitis that can complicate the diagnostic process, especially in patients with complex medical histories. This case report details a 39‐year‐old woman with situs inversus totalis, Kartagener syndrome, and hypothyroidism, who presented to the emergency department with intermittent petechiae, purpura in the lower limbs, and fever following a trip to a malaria‐endemic region. Initial investigations suggested an infectious etiology, but extensive testing for malaria and other infections returned negative results. A transition to autoimmune disease assessment was prompted by the positive results of rheumatologic tests. Pulse doses of Methylprednisolone Sodium Succinate and Rituximab were initiated, and the treatment was continued with Prednisolone, Azathioprine, and Calcium D tablets. The patient's signs and symptoms have improved after this treatment. This case underscores the necessity of considering a comprehensive differential diagnosis and advocating for a meticulous and systematic approach in complex clinical presentations.

**Summary**

- The main clinical point in this insightful article is to be aware of all possible differential diagnoses that could arise, despite all misleading data on the patient's history.
- It is also very interesting and noteworthy that we have a patient with a rare congenital condition diagnosed with a rare disease.

## 1 Introduction

Granulomatosis with polyangiitis (GPA), also known as Wegener's granulomatosis, is a rare but potentially life‐threatening autoimmune vasculitis characterized by necrotizing granulomatous inflammation predominantly affecting the respiratory tract and kidneys. It is a member of a group of diseases known as anti‐neutrophil cytoplasmic antibody (ANCA)‐associated vasculitis, which can present with a wide range of systemic symptoms and lead to significant morbidity if not diagnosed and treated promptly [1]. It affects approximately 3 in every 100,000 people and typically occurs around the age of 45 in both males and females [2]. The diagnostic journey for GPA can be particularly challenging, as its clinical presentation often resembles infections and other inflammatory conditions, especially in individuals with a complex medical history [3]. Recognizing the interplay of underlying chronic conditions [3] and emerging autoimmune phenomena is essential for timely intervention and improved patient outcomes [1].

We herein present a patient with systemic symptoms following a recent travel to a malaria‐endemic region who has finally been diagnosed with GPA at Alzahra Medical & Educational Center of Isfahan.

## 2 Case Presentation

**FIGURE 1:**
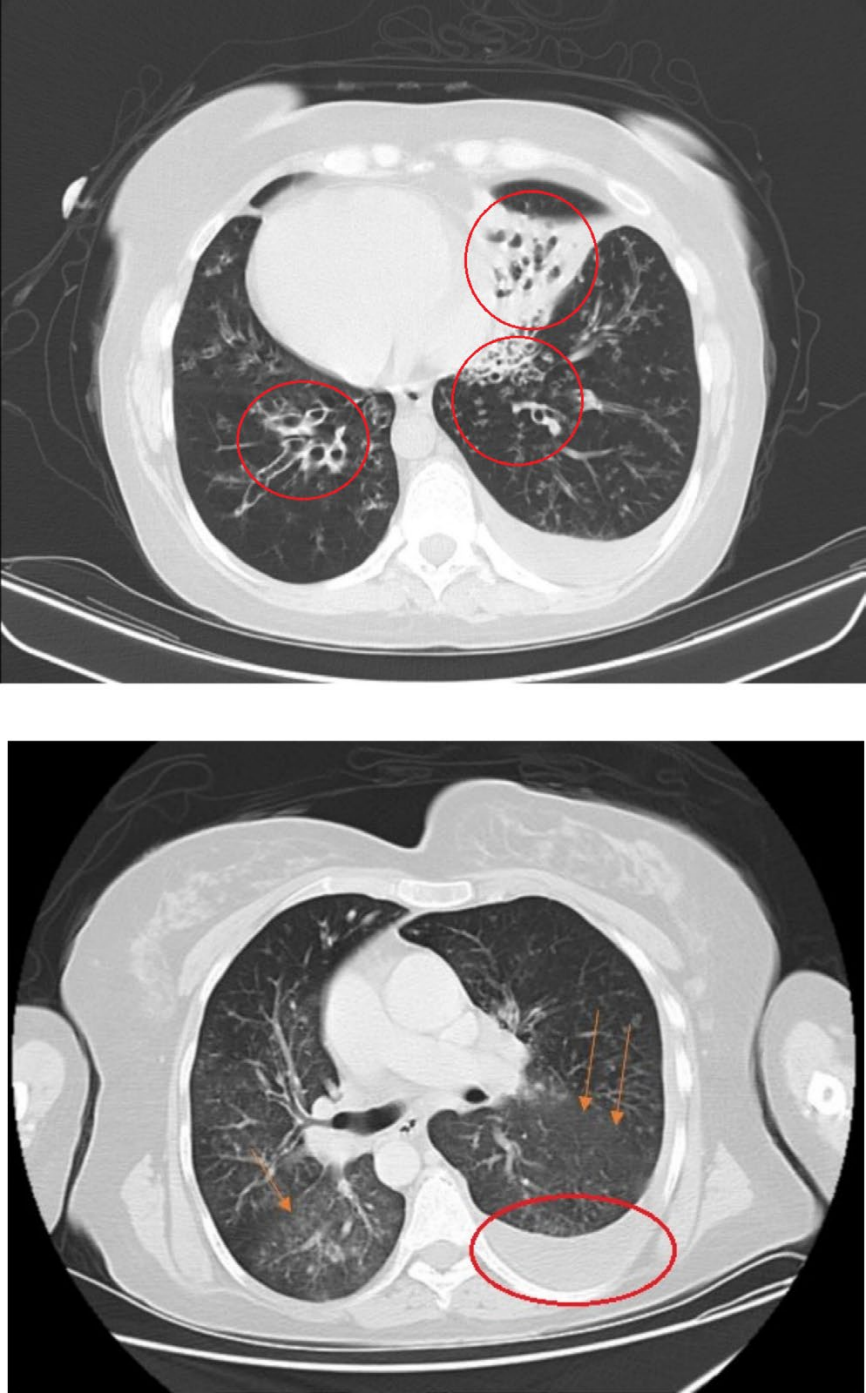
Chest M.D.C.T. Bronchiectasis in both lungs, Pleural effusion on the left side, and GGO in both lungs.

**FIGURE 2:**
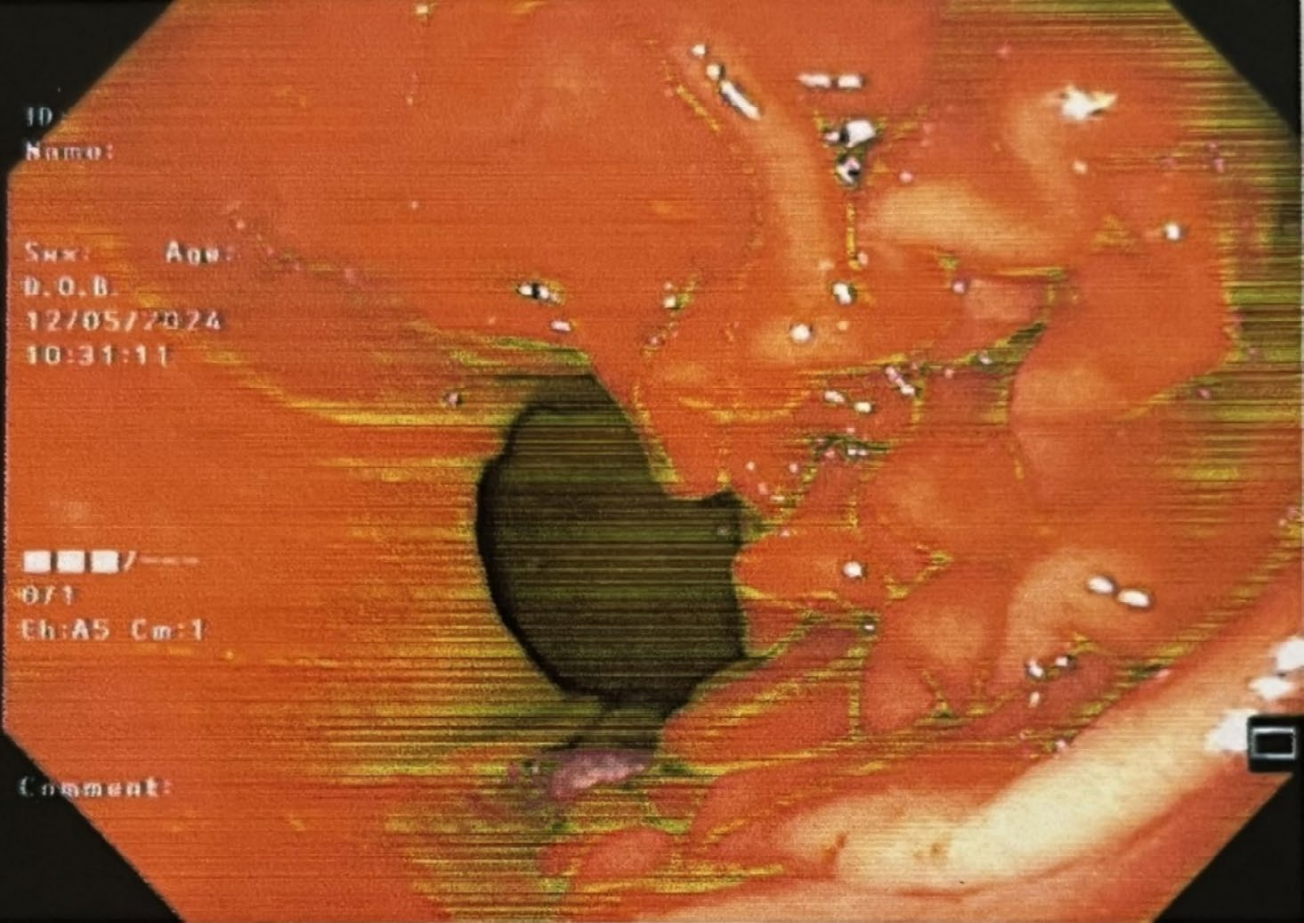
Endoscopy. Multiple erosions were seen in the body, indicating Erosive Gastropathy.

We present a case of a39‐ year‐ ld woman with situs inversus totalis and a previous history of Kartagener syndrome and hypothyroidism. She came to the emergency department with intermittent petechiae and purpura in her lower limbs, as well as intermittent high‐grade fever and true chills from 3 months ago. She was giving a history of traveling to the Malaria‐endemic region. Rashes and fever started right after her trip. She was giving a history of mild weight loss, nausea, and vomiting. Vital signs demonstrated a fever of 38.5°C, blood pressure (BP) 95/60, pulse rate (PR) 100, respiratory rate (RR) 24, and O2sat 88%. In the physical examination, we found a mild crackle in lung auscultation. Rashes were non‐blanching. Laboratories were remarkable for leukocytosis of 14.05 with left shifting and thrombocytosis of 535. ESR was 101 and CRP was 88. Serum creatinine was 1.4 at first and then rose to 1.7 in 3 days. CXR showed evidence of mild infiltration in the lower lobe of the left lung and mild pleural effusion on the left side. We tapped the pleural effusion. It had an exudative pattern in analysis. We performed a Chest M.D.C.T that showed cystic bronchiectasis (Figure 1), mild infiltration in the lower lobe of the left lung, and mild pleural effusion on the left side (Figure 1). We did an Abdominal M.D.C.T scan, which was normal. With a suspicion of Malaria, we performed PBS, but it didn't confirm Malaria. We requested blood culture, sputum culture, wright test, PPD skin test, and Widal test which all were negative. We checked viral serologic tests including HCV, and HIV which were nonreactive, and Hbs‐Ag, and Hbc‐Ab which were negative. U/A was remarkable for protein (++), blood (+++), RBC (many, 40% dysmorphic), and granular casts [1, 2]. Urine culture was negative. The stool exam, stool culture, and endoscopy were performed due to the patient's continuous nausea, vomiting, and food intolerance. The stool exam was normal, and the stool culture was negative. The endoscopy showed erosive gastropathy (Figure 2). We also ruled out endocarditis with a normal echocardiography with no evidence of vegetation. Then we changed our approach from infectious diseases that were related to her travel to rheumatologic diseases. So, we requested rheumatologic tests, and C‐ANCA (Anti PR3) and ANA, and RF were positive among them (The results are in Table 1).

**TABLE 1:** Results of autoimmune and immunology tests.

	Result	Unit	Reference interval
**Autoimmune tests**			
(F‐ANA) Fluorescent ANA. (IgG)	1:160 (Positive)	Titer	Negative: < 1:80
Pattern is DFS70			Positive: > 1:80
Anti PR3 (C‐ANCA) Fluorescent	1:40 (Positive)	Titer	Negative: < 1:20
			Borderline: 1:20
			Positive: > 1:20
Anti MPO(P‐ANCA) Fluorescent	< 1:20 (Negative)	Titer	Negative: < 1:20
			Borderline: 1:20
			Positive: > 1:20
(RF)Rheumatoid Factor IgG Quantitative	57.6 (H)	IU/ml	Normal Range: Up to 30
**Immunology test**			
(CH50) Total Hemolytic Complement	89.2	Unit	Normal Range: 70–150

## 3 Differential Diagnosis

Differential diagnoses for this case include diseases that, in addition to fever and petechial rash, are associated with the patient's travel history, elevations in ESR and CRP, pulmonary radiologic findings, positive rheumatologic tests, proteinuria, and increased creatinine levels. These differential diagnoses encompass infectious diseases such as Malaria, Dengue fever, hepatitis B and C viruses, HIV, herpes virus, Infective endocarditis, Sepsis, Mycobacterial infections, Disseminated fungal infections, Disseminated gonococcal infection, Streptococcal pneumonia with glomerulonephritis, cytomegalovirus, parvovirus B19, and parainfluenza virus, malignancies like lymphoma and pulmonary metastasis, autoimmune diseases like Systemic lupus erythematosus, Sarcoidosis, Rheumatoid arthritis, Amyloidosis, autoinflammatory diseases like Still's; drug toxicities; and rare conditions such as Thrombotic Thrombocytopenic purpura and antiphospholipid antibody syndrome, and sickle cell disease [3, 4].

## 4 Treatment Plan

At first, we had started antibiotics for her because of our suspicion of infectious diseases. Still, she had a fever for the next 3 days despite treatment with Linezolid, Meropenem, and Levofloxacin. After the positivity of the autoimmune tests, we changed our treatment. first, the patient was started on three consecutive pulse doses (1 g) of Methylprednisolone Sodium Succinate; after that, she received 1 g Rituximab, and this dose was repeated 14 days later. We continued her therapy with a Prednisolone Tablet (10 mg/three times a day), Azathioprine Tablet (daily), and Calcium D Tablet (daily). Then, a month after the second dose of Rituximab, we increased the dose of Azathioprine Tablet to 50 mg twice a day, and we slowly tapered Prednisolone. It is important to mention that we chose Rituximab among other common treatments because the patient was of reproductive age and treatments like Cyclophosphamide could lead to infertility. We also added Cotrimoxazole 400/80 mg Tablet for the prevention of Pneumocystis Carinii.

## 5 Follow Up

To date, when we write this report, the patient's signs and symptoms have improved. Serum creatinine decreased to 1.3, and urine analysis returned to normal. The level of proteinuria in the last 24‐h urine analysis was 1300 mg.

## 6 Discussion

We reported a case involving a 39‐year‐old woman who recently traveled to southern Iran, an area where malaria is endemic. She experienced intermittent petechiae on her lower limbs and suffered from a high‐grade fever. After eliminating all potential differential diagnoses and confirming her rheumatologic tests, including positive CANCA (anti‐PR3), we concluded that granulomatosis with polyangiitis (GPA) was the final diagnosis.

Antineutrophil cytoplasmic antibody ANCA‐associated vasculitis (AAV) encompasses a group of multisystem disorders that cause inflammation in the small blood vessels. This group includes granulomatosis with polyangiitis (GPA), microscopic polyangiitis (MPA), and eosinophilic granulomatosis with polyangiitis (EGPA) [5].

According to the 2022 American College of Rheumatology/European Alliance of Associations for Rheumatology (ACR/EULAR) classification criteria for GPA, only after a diagnosis of small‐ or medium‐vessel vasculitis has been confirmed and all possible vasculitis mimics have been ruled out should the criteria be utilized. (5)The classification criteria have 10 items, and a total score of ≥ 5 indicates a diagnosis of granulomatosis. In terms of laboratory, imaging, and biopsy criteria, the following apply: a positive test for cytoplasmic antineutrophil cytoplasmic antibodies (CANCA) or antiproteinase 3 (anti‐PR3) antibodies gets +5 scores [5]. Our patient got this 5 score, so we started treatment for her based on treatment guidelines for GPA.

According to the 2021 American College of Rheumatology/Vasculitis Foundation (ACR/VF) guideline for the management of antineutrophil cytoplasmic antibody‐associated vasculitis, including granulomatosis with polyangiitis (GPA), all recommendations for GPA management are conditional due to varying levels of evidence [6].

According to the ACR/VF guidelines, remission induction is divided into severe and non‐severe diseases. Severe disease is characterized by life‐threatening or organ‐threatening symptoms, which can include both multi‐organ issues and localized manifestations. Active disease is the presence of new, ongoing, or worsening signs and/or symptoms associated with GPA, which are not linked to previous damage [6].

Our patient was compatible with severe and active disease, and the ACR recommends treatment with Rituximab over Cyclophosphamide and a reduced‐dose glucocorticoid regimen for active severe cases. We employed RTX and glucocorticoid. Azathioprine was added to treatment for remission maintenance [6]. We also used Cotrimoxazole to administer prophylaxis against Pneumocystis jirovecii pneumonia as it is recommended.

These guidelines are intended to enhance patient outcomes and inform clinical decision‐making, especially in cases of treatment resistance and recovery strategies.

Untreated GPA can be a deadly condition, primarily because renal failure stemming from kidney involvement worsens recovery chances. However, treatment for GPA has significantly improved survival rates, achieving remission in over 90% of cases, especially among individuals without severe renal impairment. In contrast, the disease typically progresses swiftly and lethally when ignored, with 82% of patients succumbing within a year. Therefore, a timely and precise diagnosis of GPA is crucial to enhance the prognosis [7].

## 7 Conclusion

The diagnostic process for GPA can be quite difficult because its symptoms often mimic those of infections and other inflammatory diseases, particularly in people with complicated medical histories [3]. Our patient had a history of traveling to malaria‐endemic regions, which misled us in our approach to limb petechiae and fever, and it made our case noteworthy to report. However, further tests led to the diagnosis of GPA.

## Author Contributions

**Hanie Forouzandeh:** data curation, project administration, supervision, writing – original draft. **Ahmadreza Rajabi:** data curation, writing – original draft. **Abbas Ali Torfeh Esfahani:** conceptualization, project administration, supervision, writing – review and editing. **Farzin Khorvash:** conceptualization, writing – review and editing. **Mansoor Karimifar:** conceptualization, writing – review and editing.

## Ethics Statement

The authors have nothing to report.

## Consent

Written informed consent was obtained from all individual participants included in the study.

## Conflicts of Interest

The authors declare no conflicts of interest.

## Data Availability

Data sharing is not applicable to this article as no new data were created or analyzed in this study.
